# Estimativa de Brasileiros em Prevenção Secundária de Eventos Cardiovasculares que Não Atingem a Meta de LDL Colesterol com Tratamento Hipolipemiante

**DOI:** 10.36660/abc.20240617

**Published:** 2025-07-31

**Authors:** Andressa Braga, Marisa Santos, Carlos Magliano, Katia Senna, Bernardo Tura, Ione Oliveira

**Affiliations:** 1 Instituto Nacional de Cardiologia Núcleo de Avaliação de tecnologias em saúde Rio de Janeiro RJ Brasil Instituto Nacional de Cardiologia Núcleo de Avaliação de tecnologias em saúde, Rio de Janeiro, RJ – Brasil; 2 ASAS Valor em Saúde Rio de Janeiro RJ Brasil ASAS Valor em Saúde, Rio de Janeiro, RJ – Brasil; 3 Instituto Nacional de Cardiologia Núcleo de bioestatística e bioinformática Rio de Janeiro RJ Brasil Instituto Nacional de Cardiologia Núcleo de bioestatística e bioinformática, Rio de Janeiro, RJ – Brasil; 4 Novartis Biociências AS São Paulo SP Brasil Novartis Biociências AS, São Paulo, SP – Brasil

**Keywords:** Doenças Cardiovasculares, Prevenção Secundária, Hipolipemiantes, Estimativas Populacionais

## Abstract

**Fundamento:**

A Diretriz Brasileira de Dislipidemias de 2017 recomenda uma meta de lipoproteína de baixa densidade (LDL-c) < 50mg/dL para pacientes em prevenção secundária de eventos cardiovasculares, e tratamento com estatina e adição de ezetimiba, se necessário. Em pacientes que não atingem essa meta, é indicada farmacoterapia adicional.

**Objetivo:**

Estimar a população brasileira em prevenção secundária que não atinge a meta de LDL-c com o tratamento hipolipemiante em 2024.

**Método:**

Este estudo combinou dados populacionais do Sistema Único de Saúde (SUS) e da saúde suplementar, dados epidemiológicos e o método Delphi, com a participação de 29 especialistas na primeira rodada e 24 na segunda, para estimar o tamanho da população em prevenção secundária que não atinge a meta de LDL-c.

**Resultados:**

A população em prevenção secundária foi estimada em 5,8 milhões no sistema de saúde público e 1,2 milhão no sistema de saúde suplementar. Aproximadamente um milhão de pacientes assistidos no sistema de saúde público e 150 mil assistidos na saúde suplementar não devem atingir a meta de LDL-c com o tratamento hipolipemiante oral.

**Conclusão:**

Entre 9% e 19% dos pacientes em prevenção secundária não alcançam a meta recomendada de LDL-c, tornando-se potenciais candidatos para terapias adicionais de redução de LDL-c.

**Figure:**
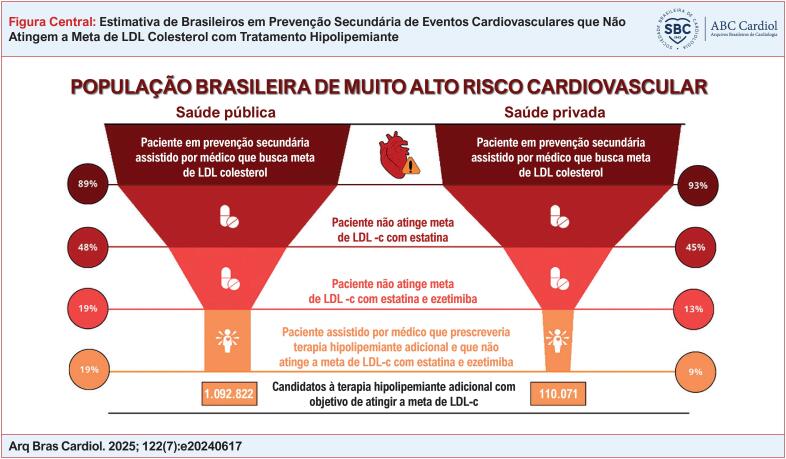


## Introdução

As doenças cardiovasculares representam a principal causa de óbito prematuro no mundo, resultando em perda da qualidade de vida, e em impactos econômicos e sociais significativos.^1 , 2^ A dislipidemia desempenha um papel importante na patogênese da aterosclerose, sendo considerada fator de risco para as doenças cardiovasculares^3 , 4^ e o seu tratamento visa redução de eventos cardiovasculares.^1 , 2^ Estima-se que 14,6% da população brasileira tenha níveis elevados da lipoproteína de baixa densidade (LDL-c).^2 , 4^

Indivíduos com eventos cardiovasculares prévios ou com diagnóstico de doença aterosclerótica são classificados como em risco cardiovascular muito alto e a prevenção de eventos nesse subgrupo é denominada prevenção secundária.^5^ A diretriz de dislipidemias e prevenção de aterosclerose da Sociedade Brasileira de Cardiologia (SBC) recomenda, para os pacientes com muito alto risco cardiovascular, a meta de LDL-c<50mg/dL.^5^ A prevenção secundária inclui estatina, associada ou não à ezetimiba.^5^ Esse tratamento leva à uma redução do LDL-c entre 45% e 60%, mas pode ser insuficiente para alcançar a meta preconizada,^3 , 6 , 7^ e terapias adicionais podem ser indicadas.^3^

No Brasil, a assistência à saúde é fornecida tanto pelo Sistema Público de Saúde (SPS), quanto pela Saúde Suplementar (SS).^8 - 10^ O SPS é representado pelo Sistema Único de Saúde (SUS), que oferece atendimento universal e gratuito, incluindo a distribuição de medicamentos.^8 , 10^ Já a SS, regulada pela Agência Nacional de Saúde (ANS), atende 25% da população, que também pode acessar o SUS.^9 , 11^

Há pouca informação sobre o percentual de pacientes em prevenção secundária que atingem a meta de LDL-c com o tratamento hipolipemiante no Brasil.^3 , 12^ Diante desta lacuna, este estudo visa estimar a população brasileira em prevenção secundária que não atinge a meta de LDL-c com estatina e ezetimiba no SPS e na SS.

## Métodos

Foram integrados dois caminhos metodológicos distintos: painel Delphi e dados populacionais. No painel Delphi, o objetivo foi identificar a conduta terapêutica adotada por cardiologistas no tratamento da dislipidemia em pacientes em prevenção secundária no SPS e na SS. Foram incluídos cardiologistas com especialização há mais de um ano, atuantes em ambulatórios do SPS ou da SS. Excluíram-se médicos com conflitos de interesse com a indústria farmacêutica ou que atuavam em ambulatórios exclusivos de dislipidemia, por potencialmente adotarem condutas que podem não refletir a prática do cardiologista geral.

**Figura 1:**
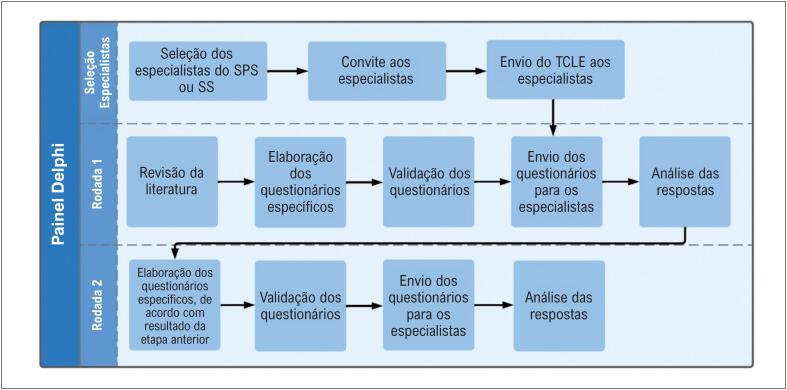
Etapas do Painel Delphi. TCLE: Termo de Consentimento Livre e Esclarecido; SPS: Sistema Público de Saúde; SS: Sistema de Saúde Suplementar. Validação do questionário: Processo metodológico que analisa o conteúdo e sua compreensão, realizado por cardiologistas externos ao painel Delphi.

O painel Delphi seguiu as etapas descritas na Figura 1 . Esse método estruturado^13^ facilita a convergência de opiniões, com rodadas de questionários e *feedback* , enquanto o anonimato permite contribuições independentes,^14 - 16^ sendo capaz de promover consenso em contextos com evidências limitadas.^13^ Os especialistas foram selecionados por amostragem não probabilística.^15^ Embora não haja um número padrão de participantes, recomenda-se entre seis e 20, para estabilidade dos resultados, com uma taxa de resposta mínima de 70% entre rodadas.^15 , 16^ Neste painel, foram realizadas duas rodadas, com consenso definido como concordância superior a 70%. Cada subgrupo deveria ter no mínimo sete participantes. Foram convidados 32 especialistas para compensar possíveis desistências. O contato foi realizado por telefone, mensagem ou e-mail. Os cardiologistas indicados pelos pesquisadores poderiam indicar outros cardiologistas (amostragem em bola de neve). Os formulários da primeira e segunda rodada foram desenvolvidos no aplicativo *Google Forms* e enviados aos participantes de cada subgrupo simultaneamente e respondidos de forma virtual e assíncrona em até 15 dias. Os painéis com os especialistas que atuam no SPS e na SS foram conduzidos entre novembro e dezembro de 2022 e entre junho e julho de 2023.

Fonte: Elaboração própria pelos autores.

A população em prevenção secundária atendida no SPS foi estimada a partir da identificação dos procedimentos cardiovasculares no Sistema de Gerenciamento da Tabela de Procedimentos, Medicamentos e OPM (Órteses, Próteses e Meios Auxiliares de Locomoção) do SUS (SIGTAP)^17^ relacionados diretamente à doença aterosclerótica ( Suplemento ). A incidência dos procedimentos selecionados foi calculada a partir dos dados do Sistema de Informação Hospitalar (SIH) de 2019^18^ e associada por meio do Cartão Nacional de Saúde (CNS) ao Sistema de Informação sobre Mortalidade (SIM),^19^ para identificar aqueles que realizaram o procedimento e evoluíram a óbito no mesmo ano. O ano de 2019 foi escolhido em função da epidemia de covid-19, que alterou o padrão de internações nos anos seguintes.^2^ Pacientes com mais de um procedimento no período foram contabilizados apenas uma vez, com base no número do CNS.

A estimativa da população em prevenção secundária na SS foi realizada com base na demanda por procedimentos relacionados à doença cardiovascular do painel D-TISS^20^ em 2019. Devido à indisponibilidade de dados de mortalidade nesta base, foi assumida a taxa de mortalidade da base SIM (12,2%). Assumiu-se que cada procedimento representaria um paciente em prevenção secundária. Foram selecionados os códigos TUSS (Terminologia Unificada da SS) relacionados diretamente à doença aterosclerótica ( Suplemento ), desconsiderando os procedimentos da aorta ascendente até aorta descendente antes da origem das artérias renais e a revascularização miocárdica em cardiopatias congênitas, por não serem considerados relacionados à aterosclerose.

Adicionalmente, adotou-se o pressuposto de que os indivíduos que realizaram pelo menos um procedimento cardiovascular em 2019 seriam equivalentes à incidência da doença. Com base no estudo Estatística Cardiovascular Brasil 2021,^21^ que forneceu as razões entre casos incidentes e prevalentes de acidente vascular cerebral (1/11,4) e infarto miocárdico (1/17,5), foi calculada uma razão sumarizada de incidência/prevalência para a população em prevenção secundária. Essa razão sumarizada, obtida por meio da média ponderada das razões específicas, utilizando a frequência dos eventos como pesos, foi aplicada para estimar a prevalência de pacientes em prevenção secundária a partir da incidência. Esses valores foram atualizados de 2019 para 2024, aplicando a taxa média de crescimento populacional do Brasil de 1,0075 para o SPS^22^ e de 0,29 para a SS.^23^

Os dados foram analisados utilizando Excel 2019^® 24^ e R versão 4.2.2.^25^ Os resultados do painel Delphi foram apresentados como porcentagens e contagens de frequência. As estimativas populacionais foram calculadas com base na média, considerado o estimador de máxima verossimilhança. Como a análise não teve o objetivo de testar hipóteses, não foram calculados valores de p ou níveis de significância estatística. Para avaliar as incertezas, foi realizada uma análise de sensibilidade probabilística com mil simulações de Monte Carlo, cujos resultados foram sumarizados por medidas de dispersão (mediana e intervalo interquartil).

### Aspectos éticos

A pesquisa seguiu as orientações da Resolução n°510/2016^26^ e nº466/12^27^ do Conselho Nacional de Saúde. Ressalta-se a isenção da necessidade de avaliação pelo sistema CEP/CONEP, por se caracterizar como "aprofundamento teórico de situações que emergem espontânea e contingencialmente na prática profissional, desde que não revelem dados que possam identificar o sujeito", conforme Resolução n°510/2016.^26^ As identidades dos especialistas do Painel Delphi foram mantidas confidenciais e apenas os pesquisadores principais tiveram acesso às informações que foram anonimizadas.

## Resultados

### Painel Delphi

Foram convidados 65 especialistas, dos quais 42 aceitaram participar, 13 foram excluídos (um por conflito de interesse, 11 por não atuarem em ambulatório ou consultório de cardiologia no SPS ou SS, e um por tempo de especialização inferior a um ano), resultando em 29 (69%) especialistas incluídos. As características dos especialistas incluídos estão na Tabela 1 .

**Tabela 1:** Rodada 1 – Característica dos entrevistados

Característica	Descrição	Total (N=29) n (%)	SPSǂ (n=12) n (%)	SS (n=17) n (%)
Cidade de atuação do especialista	Rio de Janeiro	23(79%)	8(67%)	15(88%)
	Campos dos Goytacazes	1(3%)	1(8%)	NM
	Macaé	1(3%)	1(8%)	NM
	Nova Friburgo	1(3%)	NM	1(6%)
	Ribeirão Preto	1(3%)	1(8%)	NM
	São Paulo	1(3%)	1(8%)	NM
	Atendimento remoto	1(3%)	NM	1(6%)
Tipo de ambulatório *	SPS	13(45%)	12(100%)	1(6%)
	Saúde suplementar	28(97%)	11(91,6%)	17(100%)

* em que o cardiologista assiste pacientes em prevenção secundária de evento cardiovascular;

SPSǂ: Subgrupo de especialistas do painel Delphi referente ao Sistema Público de Saúde;

SS: Subgrupo de especialistas do painel Delphi referente a saúde suplementar;

NM: Alternativa não mencionada.

Fonte: Elaboração própria pelos autores.

O painel Delphi permitiu identificar a conduta dos especialistas em relação às metas de LDL-c, os medicamentos prescritos, o percentual de pacientes que atingem a meta com estes medicamentos e o número de especialistas que prescreveriam terapia adicional com o objetivo de alcançar a meta de LDL-c.

O tratamento do paciente em prevenção secundária com estatina e a associação de ezetimiba foram consenso entre os especialistas na primeira rodada ( Tabela 2 ).

**Tabela 2:** Rodada 1 – Tratamento hipolipemiante na prevenção secundária de eventos cardiovasculares

Item	Descrição	Total (N=29) n (%)	SPSǂ (N=12) n (%)	SS (N=17) n (%)
Prescrição inicial *	Rosuvastatina	14(48%)	3(25%)	11(61%)
	Atorvastatina	12(41%)	5(42%)	7(39%)
	Sinvastatina	6(21%)	6(50%)	NM
	Estatina (não especificada)	1(3%)	1(8%)	NM
% pacientes tratados com estatina ‡‡	Todos	11(38%)	1(8%)	10(59%)
	>80%	11(38%)	11(92%)	NM
	>70%	1(3%)	NM	1(6%)
	>40% e <60%	3(10%)	NM	3(18%)
	>30% e <40%	3(10%)	NM	3(18%)
% de pacientes que atinge meta com estatina 1	<20%	1(4%) ▲	NM	1(6%) ♦
	>20% e <40%	7(26%) ▲	3(27%) ●	4(25%) ♦
	>40% e <60%	7(26%) ▲	3(27%) ●	4(25%) ♦
	>60% e <80%	12(44%) ▲	5(45%) ●	7(44%) ♦
Inicia outro hipolipemiante? †	Sim	24(83%) ††	11(92%) ††	13(76%) ††
	Não	5(17%)	1(8%)	4(24%)
Qual hipolipemiante?	Associa ezetimiba	22(92%) †† 2	11(100%) †† 3	11(85%) †† 4
	Substitui estatina e associa ezetimiba	1(4%) 2	NM	1(8%) 4
	Substitui estatina ⸙	1(4%) 2	NM	1(8%) 4
Associa ezetimiba com estatina?	Sim	22(92%) †† 2	11(100%) †† 3	11(85%) †† 4
	Não	2(8%) 2	NM	2(15%) 4
% de pacientes que alcança meta com associação	>20% e <40%	3(13%) 2	1(9%) 3	2(15%) 4
	>40% e <60%	5(21%) 2	2(18%) 3	3(23%) 4
	>60% e <80%	4(17%) 2	3(27%) 3	1(8%) 4
	>80%	12(50%) 2	5(45%) 3	7(54%) 4

* Tratamento prescrito com maior frequência;

‡‡ Percentual de pacientes que o especialista prescreve estatina;

†† Consenso alcançado;

1 pacientes assistidos por especialistas que buscam meta;

▲ n=27;

● n=11;

♦ n=16;

† nos pacientes que não alcançam a meta com estatina;

⸙ suspende atorvastatina e inicia rosuvastatina;

2 n=24;

3 n=11;

4 n=13;

N =total de especialistas no painel Delphi; n= especialistas que responderam ao critério;

SPSǂ: Subgrupo de especialistas referente ao Sistema Público de Saúde;

SS: Subgrupo de especialistas referente a Saúde suplementar;

NM: alternativa não mencionada;

**LDL-c:** Lipoproteína de baixa densidade.

Fonte: Elaboração própria pelos autores.

A segunda rodada do painel Delphi foi concluída por 83% (24/29) dos especialistas. Nesta rodada, buscou-se o consenso sobre a meta de LDL-c a ser adotada em pacientes em prevenção secundária, a partir das metas mais frequentemente citadas na primeira rodada. Houve consenso para a meta de <70 mg/dL entre especialistas da SS e para a meta de <50 mg/dL entre os especialistas da SPS. Também foi consenso o percentual de pacientes em prevenção secundária tratados com estatina, além da prescrição de farmacoterapia adicional quando a meta não foi alcançada com o tratamento combinado. Estimou-se a média de pacientes que atingem a meta de LDL-c com estatina em monoterapia ou combinada com ezetimiba baseado na opinião dos especialistas ( Tabela 3 ).

**Tabela 3:** Rodada 2 – Tratamento da dislipidemia e resposta ao tratamento

Item	Descrição	Total (N=24) (%)	SPS (N=9) (%)	SS (N=15) (%)
Busca meta de LDL-c ‡	Concordo	22(92%) †	8(89%) †	14(93%) †
	Discordo	2(8%)	1(11%)	1(7%)
Meta de LDL-c <50mg/dL	Concordo	16/22(80%) † 1	8/8(100%) † 1	8/14(57%) 1
	Discordo	1/22(5%) 1	0/8(0%) 1	1/14(7%) 1
Meta de LDL-c <70mg/dL	Concordo	15/22(75%) † 1	4/8(50%) 1	11/14(79%) † 1
	Discordo	4/22(20%) 1	3/8(43%) 1	1/14(7%) 1
Prescreve estatina para todos os pacientes em prevenção secundária	Concordo	18(75%) †	6(67%)	12(80%) †
	Discordo	6(25%)	3(33%)	3(20%)
Média ponderada de pacientes que atingem meta de LDL-c (DP)	Estatina	50,5%(±20,75)	46%(±19,97%) 3	52,2%(±20,79%) 4
	Estatina + Ezetimiba	63,8%(±10,83) *	60,9%(±9,96%) * 3	71,2%(±13,66%) * 4
Prescreveria terapia adicional 1 , 2	Sim	18/22(82%)	8/8(100%)	10/14(71%)
	Não	4/22(18%)	0/8(0%)	4/14(28%)

† Consenso alcançado (definido como concordância igual ou superior a 70%);

‡ Meta de LDL-c é o objetivo do tratamento do paciente em prevenção secundária; Concordo: Concordo fortemente ou concordo; Discordo: Discordo ou Discordo fortemente;

1 Respondido apenas por especialistas que buscam meta de LDL-c;

2 Por via subcutânea, indicada para pacientes que não atingem meta com estatina e ezetimiba;

* Dentre os pacientes que não atingem meta de LDL-c com estatina;

3,4 a média ponderada de pacientes que atingem a meta de LDL-c foram calculadas com base nas metas consensuais adotadas por cada subgrupo, sendo considerada a meta de LDL-c <50 mg/dL no SPS (100% concordância) e <70 mg/dL na SS (79% concordância) para essa estimativa

N: Total de especialistas no painel Delphi

SPS: Subgrupo de especialistas do Sistema Público de Saúde

SS: Subgrupo de especialistas da Saúde Suplementar

**LDL-c:** Lipoproteína de baixa densidade

DP: Desvio Padrão.

Fonte: Elaboração própria pelos autores.

### Estimativa da população em prevenção secundária

No SPS, foram identificados 441 890 indivíduos com ao menos um evento cardiovascular em 2019. Após ajustar a mortalidade (12,2%) e o crescimento populacional,^22^ a incidência e a prevalência estimadas para 2024 foram de 402 615 e 5 822 793 pacientes em prevenção secundária, respectivamente.

**Figura 2:**
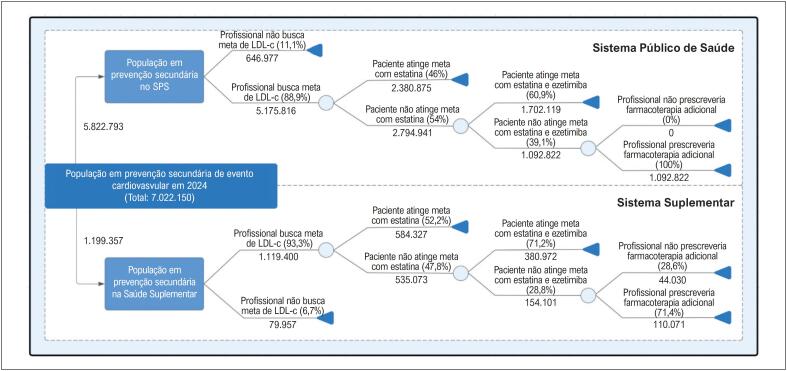
Estimativa da população candidata ao uso de farmacoterapia adicional; estimativa da população em prevenção secundária de doenças cardiovasculares com base em dados epidemiológicos e na segunda rodada do painel Delphi para a população assistida no Sistema Público de Saúde - SPS (superior) e na Saúde Suplementar - SS (inferior). Profissional busca meta de LDL-c: busca meta específica de LDL-c; Paciente não atinge meta com estatina: Paciente com indicação de associar ezetimiba a estatina; Paciente não atinge meta com estatina e ezetimiba: Paciente com indicação de terapia adicional com objetivo de reduzir o LDL-c; LDL-c: Lipoproteína de baixa densidade.

Com base no painel Delphi, calculou-se que 46% (±19,97%) dos pacientes alcançariam a meta de LDL-c com estatina, enquanto 60,9% (±9,96%) dos que não atingem essa meta em monoterapia atingiriam com estatina e ezetimiba. Assim, dos 5.175.816 pacientes atendidos por especialistas que buscam meta de LDL-c, estima-se que 2.380.875 atingiram a meta com estatina, enquanto 1.708.119 conseguiram atingi-la com estatina e ezetimiba. Portanto, 79% dos pacientes atendidos por médicos que buscam meta de LDL-c a atingiriam. Como todos esses especialistas prescreveriam terapia adicional para pacientes que não atingem a meta com estes hipolipemiantes, cerca de 1.092.822 (19%) pacientes em prevenção secundária no SPS seriam potenciais candidatos a terapia adicional para redução do LDL-c ( Figura 2 ).

Fonte: Elaboração própria pelos autores.

Na SS, foram identificados 81 796 procedimentos com base no painel D-TISS. Após ajuste da mortalidade e do crescimento populacional,^23^ a incidência e a prevalência de indivíduos com doença aterosclerótica em prevenção secundária em 2024 foram estimadas em 82 929 e em 1 199 357, respectivamente.

Segundo o painel Delphi, 52,2% (±20,79%) dos pacientes alcançariam a meta de LDL-c com estatinas e 71,2% (±13,66%) dos que não atingem essa meta em monoterapia alcançariam com estatina e ezetimiba. Portanto, entre os 1 119 400 pacientes atendidos por especialistas que buscam meta de LDL-c, estima-se que 584.327 atingiram a meta com estatina e 380 972 atingiriam a meta com estatina e ezetimiba. No total, 86% dos pacientes atendidos por médicos que buscam a meta de LDL-c, a alcançariam. Entretanto, 71,4% dos especialistas prescreveriam terapia adicional para os 154 101 pacientes que não atingissem a meta com estatina e ezetimiba, resultando em 110.071 potenciais candidatos à terapia adicional para redução do LDL-c em 2024 na SS ( Figura 2 ).

**Figura 3:**
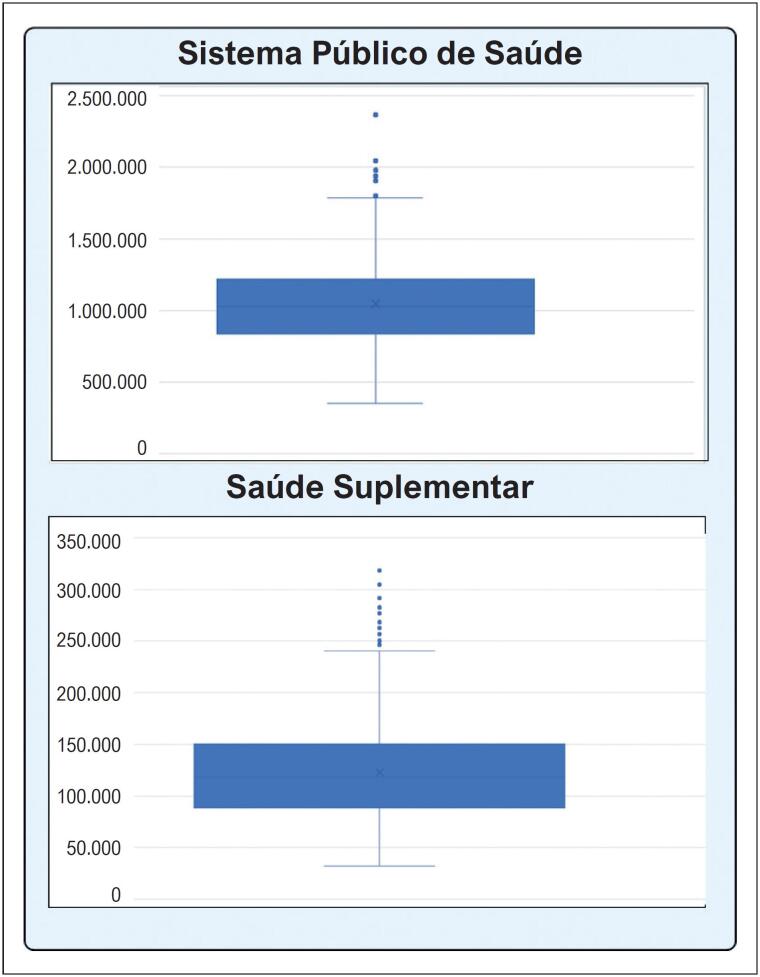
Candidatos a farmacoterapia adicional para redução do LDL-c; estimativa da população em prevenção secundária de eventos cardiovasculares atendidos por especialistas que buscam meta de LDL-c e não atingem a meta com estatina e ezetimiba, considerados potenciais candidatos a farmacoterapia adicional para redução do LDL-c, de acordo com dados epidemiológicos e painel Delphi. LDL-c: Lipoproteína de baixa densidade.

A análise de sensibilidade probabilística, considerando os parâmetros descritos na Tabela 4 , evidenciou que a mediana e intervalo interquartil da estimativa da população candidata à terapia hipolipemiante adicional é de 1 046 182 (838.337-1.218.850) no SPS e de 118 301 (88.507-150.600) na SS ( Figura 3 ).

**Tabela 4:** Parâmetros para estimar a população em prevenção secundária 1 candidata à farmacoterapia adicional

Grupo	Característica	Est. Pontual	Lim. Inferior	Lim. Superior	Dist.	Fonte
SPS	População em prevenção secundária 2024	5.822.793	4.658.234	6.987.352	Gama	SIH 2019
	Percentual de especialistas que busca meta de LDL-c	88,9%	71,1%	100,0%	Beta	Delphi
	Percentual de pacientes que atinge a meta com estatina	46,0%	26,0%	66,0%	Beta	Delphi
	Percentual de pacientes que atinge a meta com ezetimiba	60,9%	50,9%	70,9%	Beta	Delphi
	Percentual de especialistas que prescreve farmacoterapia adicional *	100%	91,7%	100%	Aleatório	Delphi
SS	População em prevenção secundária 2024	1.199.357	959.486	1.439.228	Gama	D-TISS 2019
	Percentual de especialistas que busca meta de LDL-c	93,3%	74,7%	100,0%	Beta	Delphi
	Percentual de pacientes que atinge a meta com estatina	52,2%	31,4%	73,0%	Beta	Delphi
	Percentual de pacientes que atinge a meta com ezetimiba	71,2%	57,54%	84,86%	Beta	Delphi
	Percentual de especialistas que prescreve medicamento adicional *	71,4%	66,7%	93,3%	Beta	Delphi

* com objetivo de reduzir LDL-c;

1 de novos eventos cardiovasculares, que não atingem a meta de LDL-c com estatinas e ezetimiba

Lim.: Limite; Est.: Estimativa; Dist.: Distribuição; LDL-c: Lipoproteína de baixa densidade;

SPS: Sistema Público de Saúde;

SS: Saúde suplementar.

Fonte: Elaboração própria pelos autores.

Fonte: Elaboração própria pelos autores.

## Discussão

Os resultados do painel Delphi realizado neste estudo identificou que dois especialistas (7%) não tinham como objetivo alcançar meta de LDL-c, um de cada subgrupo. Dentre os demais, a meta de LDL-c<50mg/dL, recomendada pela SBC,^3^ era o objetivo dos especialistas no SPS e de 57% na SS, nesta, o consenso foi obtido para meta inferior a 70mg/dL.

Contrariamente às recomendações da SBC, o Protocolo Clínico e Diretrizes Terapêuticas de Dislipidemia do Ministério da Saúde recomenda terapia hipolipemiante agressiva, sem meta específica nos pacientes em prevenção secundária.^4^ Essas divergências também são observadas na literatura médica.^28^ Entre 2015 e 2020, estudos com pacientes de muito alto risco utilizaram com maior frequência a meta de referência de LDL-c<70mg/dL,^29 - 38^ enquanto aqueles publicados após 2021 a meta foi reduzida para 55mg/dL.^39 - 41^

O painel Delphi indicou que a estatina é prescrita para mais de 80% dos pacientes em prevenção secundária, semelhante ao estudo REACT,^42^ que evidenciou a prescrição de estatina para 77,7% dos pacientes ambulatoriais em prevenção secundária no SPS ou na SS,^42^ entretanto superior ao número de pacientes de alto risco cardiovascular que informaram utilizar o hipolipemiante (55,2%) no início do estudo ELSA-Brasil.^43^

Em relação à estatina prescrita, um estudo retrospectivo conduzido em um hospital brasileiro evidenciou que a sinvastatina (77,6%) e a atorvastatina (22,4%) foram prescritas com maior frequência para a população assistida no SPS.^44^ Neste estudo, a sinvastatina também foi mencionada com maior frequência no SPS, já na SS foi a rosuvastatina. Não foi possível estabelecer o motivo desta diferença, mas a oferta gratuita de sinvastatina e atorvastatina pelo SUS,^45^ e o custo elevado da rosuvastatina, podem justificar parte desses achados.

A diferença no percentual de pacientes que atingem a meta no SPS e na SS podem estar relacionadas a divergência das metas informadas pelos especialistas ou à potência das estatinas. Além disso, diferenças socioeconômicas e de acesso a saúde podem contribuir para um maior controle do LDL-c na SS, como evidenciado no estudo ELSA-Brasil, onde o controle de LDL-c foi mais frequente na SS (62,4%) em comparação ao SPS (45,6%).^43^ Contudo, ao analisar especificamente os pacientes em prevenção secundária, um estudo retrospectivo estimou que apenas 7,4% dos pacientes assistidos no SPS apresentaram níveis de LDL-c<50mg/dL e 28,9% LDL-c<70mg/dL.^12^

Dentre os especialistas que buscam meta de LDL-c, todos no SPS e 71,4% na SS prescreveriam farmacoterapia adicional aos pacientes que não atingiram a meta recomendada com o tratamento hipolipemiante, resultado semelhante ao observado em outro estudo, no qual 80% dos especialistas prescreveriam terapia adicional.^46^ A prescrição mais frequente no SPS pode refletir a complexidade clínica dos pacientes atendidos, além da distribuição gratuita de medicamentos. Já na SS, o custo elevado dos medicamentos usados pelos pacientes em prevenção secundária pode ser considerado um fator limitante.

O percentual de pacientes candidatos à terapia adicional na SS é consistente com as estimativas de Cannon et al.,^47^ que estimaram que 86% dos pacientes em prevenção secundária tratados com estatinas, isoladas ou associadas à ezetimiba, alcançariam LDL-c <70mg/dL, enquanto 14% necessitariam de terapia adicional.^47^ Já Virani e et al.^48^ estimaram que 24,5% dos pacientes em prevenção secundária, tratados com estatinas e ezetimiba, seriam candidatos à terapia adicional, percentual semelhante a encontrada no SPS.^48^ Esses dados reforçam que, embora estatina e ezetimiba sejam eficazes para reduzir o LDL-c, muitos pacientes podem se beneficiar de terapias adicionais para alcançar a meta de LDL-c recomendada pela diretriz da SBC.^3^ Atualmente, no Brasil, três tecnologias estão registradas para essa indicação: alirocumabe, evolocumabe,^3^ e inclisirana.^49^

Dentre as limitações do estudo, destaca-se a impossibilidade de estimar o percentual de pacientes que atingem a meta por tipo de estatina, visto que a potência dos fármacos diverge. A ausência de entrevistas com pacientes impediu a avaliação da aderência ao tratamento hipolipemiante e da disposição para utilizar terapias adicionais subcutâneas. Além disso, assumir que o procedimento equivale a um evento cardiovascular (incidência) pode subestimar a população em prevenção secundária, pois pacientes sem eventos agudos, mas diagnosticados ambulatorialmente (ex.: angina estável), não foram contabilizados. Contudo, considerando o caráter complexo desses eventos, o impacto provavelmente é pequeno. A validade externa é restrita, pela predominância de especialistas do Rio de Janeiro, que limita a extrapolação nacional dos resultados, devido a variações regionais nas condutas médicas e metas adotadas. Outras limitações inerentes ao painel Delphi incluem o uso de amostra de conveniência, o tamanho da amostra e a redução no número de participantes entre as rodadas. Apesar dessas limitações, estudos prospectivos que avaliem as taxas de sucesso no alcance das metas de LDL-c em populações reais, bem como análise de custo-efetividade de terapias adicionais no SUS e na SS, podem contribuir para aprimorar o manejo da dislipidemia no Brasil.

## Conclusão

A partir da análise realizada deste estudo, estima-se que, em 2024, aproximadamente seis milhões de brasileiros estarão em prevenção secundária de eventos cardiovasculares no SPS e cerca de um milhão na SS (Figura Central). Além disso, acredita-se que entre 9% e 19% desses pacientes não atingirão a meta de LDL-c recomendada pelas diretrizes médicas atuais e poderiam se beneficiar da terapia adicional para redução do LDL-c, com a finalidade de reduzir o risco de novos eventos cardiovasculares.

Disponibilidade de Dados

Os conteúdos subjacentes ao texto da pesquisa estão contidos no manuscrito.

## *Material suplementar

Para informação adicional, por favor, clique aqui


