# Supplementary material for: Estimativa de Brasileiros em Prevenção Secundária de Eventos Cardiovasculares que Não Atingem a Meta de LDL Colesterol com Tratamento Hipolipemiante
Source: Arq Bras Cardiol. 2025 Jul 31;122(7):e20240617. [Article in Portuguese] doi: 10.36660/abc.20240617 (PMC12694997; doi:10.36660/abc.20240617)
Supplement: Supplementary file 1 [file 0066-782X-abc-122-07-e20240617-Suppl01.pdf]

SUPLEMENTO 1 – Tabela S1- Lista de códigos extraídos do SIGTAP (DataSUS) utilizados na análise

Código SIGTAP	Descrição Procedimento
03.03.04.014-9	TRATAMENTO DE ACIDENTE VASCULAR CEREBRAL - AVC (ISQUEMICO OU HEMORRAGICO AGUDO)
03.03.04.030-0	TRATAMENTO DO ACIDENTE VASCULAR CEREBRAL ISQUÊMICO AGUDO COM USO DE TROMBOLÍTICO
03.03.06.001-8	TRATAMENTO DE ANEURISMA DA AORTA
03.03.06.004-2	TRATAMENTO DE CARDIOPATIA ISQUEMICA CRONICA
03.03.06.006-9	TRATAMENTO DE CHOQUE CARDIOGENICO
03.03.06.019-0	TRATAMENTO DE INFARTO AGUDO DO MIOCÁRDIO
03.03.06.020-4	TRATAMENTO DE INSUFICIENCIA ARTERIAL C/ ISQUEMIA CRÍTICA
03.03.06.028-0	TRATAMENTO DE SINDROME CORONARIANA AGUDA
04.06.01.070-6	INFARTECTOMIA / ANEURISMECTOMIA ASSOCIADA OU NÃO A REVASCULARIZAÇÃO MIOCÁRDICA
04.06.01.081-1	PLÁSTICA VALVAR C/ REVASCULARIZAÇÃO MIOCÁRDICA
04.06.01.092-7	REVASCULARIZAÇÃO MIOCÁRDICA C/ USO DE EXTRACÓRPOREA
04.06.01.093-5	REVASCULARIZAÇÃO MIOCÁRDICA C/ USO DE EXTRACÓRPOREA (C/ 2 OU MAIS ENXERTOS)
04.06.01.094-3	REVASCULARIZAÇÃO MIOCÁRDICA S/ USO DE EXTRACORPÓREA
04.06.01.095-1	REVASCULARIZAÇÃO MIOCÁRDICA S/ USO DE EXTRACORPÓREA (C/ 2 OU MAIS ENXERTOS)

Código SIGTAP	Descrição Procedimento
04.06.01.120-6	TROCA VALVAR C/ REVASCULARIZAÇÃO MIOCÁRDICA
04.06.02.031-0	PONTE AXILO-BIFEMURAL
04.06.02.032-9	PONTE AXILO-FEMURAL
04.06.02.033-7	PONTE DE RAMOS DOS TRONCOS SUPRA-AORTICOS
04.06.02.035-3	PONTE-TROMBOENDARTERECTOMIA AORTO-FEMURAL
04.06.02.036-1	PONTE-TROMBOENDARTERECTOMIA AORTO-ILÍACA
04.06.02.037-0	PONTE-TROMBOENDARTERECTOMIA DE CARÓTIDA
04.06.02.038-8	PONTE-TROMBOENDARTERECTOMIA ILIACO-FEMURAL
04.06.02.041-8	REVASCULARIZAÇÃO DE ARTÉRIAS VISCERAIS
04.06.02.042-6	REVASCULARIZAÇÃO DO MEMBRO SUPERIOR
04.06.02.043-4	- REVASCULARIZAÇÃO POR PONTE / TROMBOENDARTERECTOMIA DE OUTRAS ARTERIAS DISTAIS
04.06.02.044-2	REVASCULARIZAÇÃO POR PONTE / TROMBOENDARTERECTOMIA FEMURO-POPLÍTEA DISTAL
04.06.02.045-0	REVASCULARIZAÇÃO POR PONTE / TROMBOENDARTERECTOMIA FEMURO-POPLÍTEA PROXIMAL
04.06.02.058-2	TROCA DE AORTA DESCENDENTE (INCLUI ABDOMINAL)
04.06.03.001-4	ANGIOPLASTIA CORONARIANA
04.06.03.002-2	ANGIOPLASTIA CORONARIANA C/ IMPLANTE DE DOIS STENTS

Código SIGTAP	Descrição Procedimento
04.06.03.003-0	ANGIOPLASTIA CORONARIANA COM IMPLANTE DE STENT
04.06.03.004-9	ANGIOPLASTIA CORONARIANA PRIMÁRIA
04.06.03.005-7	ANGIOPLASTIA COM IMPLANTE DE DUPLO STENT EM AORTA/ARTERIA PULMONAR E RAMOS
04.06.03.006-5	ANGIOPLASTIA EM ENXERTO CORONARIANO
04.06.03.007-3	ANGIOPLASTIA EM ENXERTO CORONARIANO (COM IMPLANTE DE STENT)

SUPLEMENTO 1 – Tabela S2 - Procedimentos consultados no painel D-TISS referente a saúde suplementar

Código TUSS	Descrição	Quantidade
30903025	Revascularização do miocárdio	4410
30903033	Revascularização do miocárdio + cirurgia valvar	470
30906016	Aneurisma de aorta abdominal infra-renal	485
30906040	Aneurisma de artérias viscerais	69
30906059	Aneurisma de axilar, femoral, poplítea	315
30906067	Aneurisma de carótida, subclávia, ilíaca	275
30906113	Angioplastia transluminal transoperatória - por artéria	12160
30906121	Artéria hipogástrica - unilateral - qualquer técnica	145
30906130	Artéria mesentérica inferior - qualquer técnica	24
30906148	Artéria mesentérica superior - qualquer técnica	65
30906156	Artéria renal bilateral revascularização	61
30906180	Endarterectomia aorto-ilíaca	79
30906199	Endarterectomia carotídea - cada segmento arterial tratado	2113
30906202	Endarterectomia ilíaco-femoral	1102
30906229	Ponte aorto-bifemoral	69
30906237	Ponte aorto-biilíaca	84
30906245	Ponte aorto-femoral - unilateral	76
30906253	Ponte aorto-ilíaca - unilateral	128
30906261	Ponte axilo-bifemoral	36
30906270	Ponte axilo-femoral	15
30906288	Ponte distal	841
30906296	Ponte fêmoro poplítea proximal	520
30906300	Ponte fêmoro-femoral cruzada	126
30906318	Ponte fêmoro-femoral ipsilateral	55
30906326	Ponte subclávio bifemoral	NI
30906334	Ponte subclávio femoral	1

Código TUSS	Descrição	Quantidade
30906342	Pontes aorto-cervicais ou endarterectomias dos troncos supra-aórtico	356
30906350	Pontes transcervicais - qualquer tipo	27
30906385	Arterioplastia da femoral profunda (profundoplastia)	838
30906415	Revascularização aorto-femoral-unilateral	85
30906423	Revascularização arterial de membro superior	510
30906431	Tratamento cirúrgico da isquemia cerebral	97
30910013	Aneurisma roto ou trombosado de aorta abdominal abaixo da artéria renal	73
30910021	Aneurismas rotos ou trombosados - outros	181
30910048	Aneurismas rotos ou trombosados de artérias viscerais	38
30910056	Aneurismas rotos ou trombosados de axilar, femoral, poplítea	104
30910064	Aneurismas rotos ou trombosados de carótida, subclávia, ilíaca	51
30912032	Angioplastia transluminal percutânea de múltiplos vasos, com implante	9724
30912040	Angioplastia transluminal percutânea por balão (1 vaso)	5697
30912105	Implante de stent coronário com ou sem angioplastia	17310
30912113	Infusão seletiva intravascular de enzimas trombolíticas	554
30912180	Recanalização arterial no IAM - angioplastia primária - com implante	4217
30912199	Recanalização mecânica do IAM (angioplastia primária com balão)	606
30912229	Revascularização transmiocárdica percutânea	1
30912261	Angioplastia transluminal percutânea de bifurcação e de tronco com implante de stent	3703
40813061	Angioplastia de ramo intracraniano	1258
40813070	Angioplastia de tronco supra-aórtico	1063
40813100	Angioplastia de artéria visceral - por vaso	101
40813177	Angioplastia transluminal percutânea	2569
40813193	Colocação de stent em ramo intracraniano	1280
40813207	Colocação de stent em tronco supra-aórtico	1040
40813215	Colocação de stent aórtico	22
40813258	Colocação de stent em artéria visceral por vaso	129

Código TUSS	Descrição	Quantidade
40813266	Colocação de stent para tratamento de obstrução arterial ou venosa - por vaso	3698
40813355	Colocação percutânea de stent vascular	686
40813940	Implante de endoprótese em aneurisma de aorta abdominal ou torácica com stent revestido (stent-graft)	1027
40813959	Implante de endoprótese em dissecção de aorta abdominal ou torácica com stent revestido (stent-graft)	67
40814025	Trombólise medicamentosa arterial ou venosa - por vaso	743
40814033	Trombólise medicamentosa arterial ou venosa para tratamento de isquemia mesentérica	21
40814041	Trombólise medicamentosa em troncos supra-aórticos e intracranianos	196
40814220	Trombectomia no AVC isquêmico agudo	NI
	Total de procedimentos	81796

Legenda: NI: Não informado
