# Supplementary material for: Estimativa de Brasileiros em Prevenção Secundária de Eventos Cardiovasculares que Não Atingem a Meta de LDL Colesterol com Tratamento Hipolipemiante
Source: Arq Bras Cardiol. 2025 Jul 31;122(7):e20240617. [Article in Portuguese] doi: 10.36660/abc.20240617 (PMC12694997; doi:10.36660/abc.20240617)
Supplement: Translation

# Translation: Estimate of Brazilians Under Secondary Prevention of Cardiovascular Events Who Do Not Achieve LDL Cholesterol Targets with Lipid-Lowering Therapy

**DOI:** 10.36660/abc.20240617i

**Authors:** Andressa Braga, Marisa Santos, Carlos Magliano, Katia Senna, Bernardo Tura, Ione Oliveira

**Affiliations:** 1 Rio de Janeiro RJ Brazil Instituto Nacional de Cardiologia Núcleo de Avaliação de tecnologias em saúde, Rio de Janeiro, RJ – Brazil; 2 Rio de Janeiro RJ Brazil ASAS Valor em Saúde, Rio de Janeiro, RJ – Brazil; 3 Rio de Janeiro RJ Brazil Instituto Nacional de Cardiologia Núcleo de bioestatística e bioinformática, Rio de Janeiro, RJ – Brazil; 4 São Paulo SP Brazil Novartis Biociências AS, São Paulo, SP – Brazil

**Keywords:** Cardiovascular Diseases, Secondary Prevention, Hypolipidemic Agents, Population Forecast

## Abstract

**Background:**

The 2017 Brazilian Guideline on Dyslipidemias recommends a low-density lipoprotein (LDL-c) target of <50 mg/dL for patients under secondary prevention of cardiovascular events, with statin therapy and the addition of ezetimibe if necessary. For patients who do not achieve this target, additional pharmacotherapy is indicated.

**Objectives:**

This study combined population data from the Brazilian Unified Health System (SUS) and the supplementary health system, epidemiological data, and the Delphi method, with participation from 29 specialists in the first round and 24 in the second, to estimate the size of the secondary prevention population not achieving LDL-c targets.

**Results:**

The population under secondary prevention was estimated at 5,8 million in the public health system and 1,2 million in the supplementary health system. Approximately one million patients in SUS and 150 thousand in the supplementary system are not expected to reach the LDL-c target with oral lipid-lowering therapy.

**Conclusion:**

Between 9% and 19% of patients under secondary prevention do not reach the recommended LDL-c target, making them potential candidates for additional LDL-c–lowering therapies.

## Introduction

Cardiovascular diseases are the leading cause of premature death worldwide, resulting in loss of quality of life and significant economic and social impacts.^1 , 2^Dyslipidemia plays an important role in the pathogenesis of atherosclerosis and is considered a risk factor for cardiovascular diseases;^3 , 4^its treatment aims at reducing cardiovascular events.^1 , 2^It is estimated that 14.6% of the Brazilian population has elevated levels of low-density lipoprotein (LDL-c).^2 , 4^

Individuals with previous cardiovascular events or a diagnosis of atherosclerotic disease are classified as at very high cardiovascular risk, and the prevention of events in this subgroup is called secondary prevention.^5^The Brazilian Society of Cardiology (SBC) guideline on dyslipidemia and prevention of atherosclerosis recommends an LDL-c target of <50 mg/dL for these patients.^5^Secondary prevention includes statin therapy, with or without ezetimibe.^5^This treatment leads to a reduction in LDL-c levels between 45% and 60%, but may be insufficient to reach the recommended target,^3 , 6 , 7^and additional therapies may be indicated.^3^

In Brazil, healthcare is provided both through the public health system (PHS) and through supplementary health (SH) care.^8 - 10^The PHS is represented by the Unified Health System (SUS, Sistema Único de Saúde), which offers universal and free care, including the distribution of medications.^8 , 10^SH care is regulated by the Brazilian National Supplementary Health Agency (ANS), and serves 25% of the population, which can also access the SUS.^9 , 11^

There is limited information on the percentage of patients undergoing secondary prevention who achieve the LDL-c target with lipid-lowering therapy in Brazil.^3 , 12^In light of this gap, this study aims to estimate the Brazilian population in secondary prevention who do not reach the LDL-c goal with statin and ezetimibe therapy in the PHS and SH.

## Methods

Two distinct methodological approaches were integrated: a Delphi panel and population data. The objective of the Delphi panel was to identify the therapeutic practices adopted by cardiologists in the treatment of dyslipidemia in secondary prevention patients within the PHS and SH. Cardiologists with more than one year of specialization and working in outpatient clinics of the PHS or SH were included. Physicians with conflicts of interest with the pharmaceutical industry or those working exclusively in dyslipidemia clinics were excluded, as they might adopt practices that do not reflect the general cardiologist’s clinical practice.

The Delphi panel followed the steps outlined in Figure 1 . This structured method^13^facilitates the convergence of opinions through rounds of questionnaires and feedback, while anonymity allows for independent contributions,^14 - 16^enabling consensus-building in contexts with limited evidence.^13^The experts were selected through non-probabilistic sampling.^15^Although there is no standard number of participants, a recommendation of six to 20 is generally made to ensure result stability, with a minimum response rate of 70% between rounds.^15 , 16^In this panel, two rounds were conducted, with consensus defined as agreement greater than 70%. Each subgroup was required to have at least seven participants. A total of 32 experts were invited to account for potential dropouts. Contact was made via phone, message, or email. Cardiologists recommended by researchers could refer other cardiologists (snowball sampling). The questionnaires for the first and second rounds were developed using the Google Forms application and sent simultaneously to participants of each subgroup, to be completed virtually and asynchronously within 15 days. The panels with experts from the PHS and SH were conducted between November and December 2022 and between June and July 2023.

**Figure 1:** Delphi panel stages; questionnaire validation: methodological process that analyzes the content and its comprehension, carried out by cardiologists external to the Delphi panel; PHS: public health system; SH: supplementary health.

Source: the authors.

The population under secondary prevention attending public health centers was estimated based on the identification of cardiovascular procedures in the SUS management system of the table of procedures, medications, and orthotics, prosthetics, and mobility aids (SIGTAP)^17^directly related to atherosclerotic disease (Supplement). The incidence of selected procedures was calculated using data from the 2019 Hospital Information System (SIH)^18^and associated through the National Health Card (NHC) to the Mortality Information System (SIM)^19^to identify individuals who underwent the procedure and died in the same year. The year 2019 was chosen due to the COVID-19 pandemic, which altered the pattern of hospital admissions in subsequent years.^2^Patients who underwent more than one procedure during the period were counted only once, based on their NHC number.

The estimate of the population under secondary prevention in the SH was based on the demand for procedures related to cardiovascular disease from the D-TISS^20^panel in 2019. Due to the unavailability of mortality data in this database, a mortality rate from the SIM database (12.2%) was assumed. It was also assumed that each procedure represented one patient undergoing secondary prevention. SH unified terminology codes directly related to atherosclerotic disease (Supplement) were selected, excluding procedures involving the ascending aorta to the descending aorta before the origin of the renal arteries, as well as myocardial revascularization in congenital heart diseases, since these are not considered related to atherosclerosis.

Additionally, it was assumed that individuals who underwent at least one cardiovascular procedure in 2019 were equivalent to the incidence of the disease. Based on the study Cardiovascular Statistics – Brazil 2021,^21^which provided the ratios between incident and prevalent cases of stroke (1/11.4) and myocardial infarction (1/17.5), a summarized incidence/prevalence ratio was calculated for the population under secondary prevention. This summarized ratio, obtained through the weighted average of the specific ratios using the event frequencies as weights, was applied to estimate the prevalence of patients under secondary prevention based on incidence. These values were updated from 2019 to 2024 using Brazil’s average population growth rate: 1.0075 for PHS^22^and 0.29 for SH.^23^

The data were analyzed using Excel 2019^® 24^and R version 4.2.2.^25^The results of the Delphi panel were presented as percentages and frequency counts. Population estimates were calculated based on the mean, using the maximum likelihood estimator. Since the analysis was not intended to test hypotheses, no p-values or statistical significance levels were calculated. To assess uncertainties, a probabilistic sensitivity analysis was conducted with one thousand Monte Carlo simulations, and the results were summarized using dispersion measures (median and interquartile range).

### Ethical aspects

The research followed the guidelines of Resolutions No. 510/2016^26^and No. 466/12^27^of the Brazilian National Health Council. It is important to note that the study was exempt from evaluation by the CEP/CONEP system, as it is characterized as a ‘theoretical deepening of situations that arise spontaneously and contingently in professional practice, provided that they do not reveal data that could identify the subject’, in accordance with Resolution No. 510/2016.^26^The identities of the Delphi Panel experts were kept confidential, and only the principal researchers had access to the anonymized information.

## Results

### Delphi Panel

A total of 65 experts were invited, of whom 42 agreed to participate. Thirteen were excluded (one due to a conflict of interest, 11 for not working in cardiology outpatient clinics or offices in the PHS or SH, and one for having less than one year of specialization), resulting in 29 (69%) experts included. The characteristics of the included experts are presented in Table 1 .

**Table 1:** Round 1 – Characteristics of the the interviewees

Characteristics	Description	Total (N=29) n (%)	PHSǂ (n=12) n (%)	SH (n=17) n (%)
Specialist’s city of practice	Rio de Janeiro	23(79%)	8(67%)	15(88%)
	Campos dos Goytacazes	1(3%)	1(8%)	NM
	Macaé	1(3%)	1(8%)	NM
	Nova Friburgo	1(3%)	NM	1(6%)
	Ribeirão Preto	1(3%)	1(8%)	NM
	São Paulo	1(3%)	1(8%)	NM
	Virtual care setting	1(3%)	NM	1(6%)
Outpatient clinic *	PHS	13(45%)	12(100%)	1(6%)
	SH	28(97%)	11(91.6%)	17(100%)

* where cardiologist treats patients in secondary prevention of cardiovascular events;

PHSǂ: Delphi panel subgroup of experts from the public health system;

SH: Delphi panel subgroup of experts from the supplementary health care system;

NM: not mentioned;

source: the authors.

The Delphi panel made it possible to identify the specialists’ approach regarding LDL-c targets, the prescribed medications, the percentage of patients who reach the target with these medications, and the number of specialists who would prescribe additional therapy with the aim of achieving the LDL-c target.

Treatment of patients under secondary prevention with statins and the combination with ezetimibe reached consensus among the specialists in the first round ( Table 2 ).

**Table 2:** Round 1 – Hypolipidemic treatment in secondary prevention of cardiovascular events

Item	Description	Total (N=29) n (%)	PHSǂ (n=12) n (%)	SH (n=17) n (%)
Initial prescription *	Rosuvastatin	14(48%)	3(25%)	11(61%)
	Atorvastatin	12(41%)	5(42%)	7(39%)
	Simvastatin	6(21%)	6(50%)	NM
	Statin (not specified)	1(3%)	1(8%)	NM
% patients treated with statin ‡‡	Total	11(38%)	1(8%)	10(59%)
	>80%	11(38%)	11(92%)	NM
	>70%	1(3%)	NM	1(6%)
	>40% and <60%	3(10%)	NM	3(18%)
	>30% and <40%	3(10%)	NM	3(18%)
% patients that reaches statin target 1	<20%	1(4%) ▲	NM	1(6%) ♦
	>20% and <40%	7(26%) ▲	3(27%) ●	4(25%) ♦
	>40% and <60%	7(26%) ▲	3(27%) ●	4(25%) ♦
	>60% and <80%	12(44%) ▲	5(45%) ●	7(44%) ♦
Starts another lipid-lowering agent? †	Yes	24(83%) ††	11(92%) ††	13(76%) ††
	No	5(17%)	1(8%)	4(24%)
Starts another lipid-lowering agent? †	Combines ezetimibe	22(92%) †† 2	11(100%) †† 3	11(85%) †† 4
	Replace statin and add ezetimibe	1(4%) 2	NM	1(8%) 4
	Replace statin ⸙	1(4%) 2	NM	1(8%) 4
Combines ezetimibe with statin?	Yes	22(92%) †† 2	11(100%) †† 3	11(85%) †† 4
	No	2(8%) 2	NM	2(15%) 4
% patients that reaches the target with combined therapy	>20% and <40%	3(13%) 2	1(9%) 3	2(15%) 4
	>40% and <60%	5(21%) 2	2(18%) 3	3(23%) 4
	>60% and <80%	4(17%) 2	3(27%) 3	1(8%) 4
	>80%	12(50%) 2	5(45%) 3	7(54%) 4

* Most frequently prescribed treatment;

‡‡ Percentage of patients for whom the specialist prescribes statins;

†† Consensus reached;

1 patients managed by specialists aiming for the target;

▲ n=27;

● n=11;

♦ n=16;

† for patients who do not reach the target with statins;

⸙ discontinues atorvastatin and initiates rosuvastatin;

2 n=24;

3 n=11;

4 n=13;

N = total number of specialists in the Delphi panel; n = number of specialists who responded to the criterion;

PHSǂ: Subgroup of experts from the Public Health System;

SH: Subgroup of experts from the Supplementary Health system;

NM: alternative not mentioned;

**LDL-c:** Low-density lipoprotein cholesterol,

Source: the authors.

The second round of the Delphi panel was completed by 83% (24/29) of the experts. In this round, the goal was to reach a consensus on the LDL-c target to be adopted for patients under secondary prevention, based on the most frequently cited targets from the first round. Consensus was reached for a target of <70 mg/dL among SH experts, and <50 mg/dL among PHS experts. There was also a consensus on the percentage of patients in secondary prevention treated with statins, as well as the prescription of additional pharmacotherapy when the target was not achieved with combined treatment. The average number of patients who reach the LDL-c target with statin monotherapy or in combination with ezetimibe was estimated based on the specialists’ opinions ( Table 3 ).

**Table 3:** Round 2 – Dyslipidemia treatment and response to treatment

Item	Description	Total (N=29) n (%)	PHS ^ǂ^(n=12) n (%)	SH (n=17) n (%)
Aims LDL-c target ‡	Agree	22(92%) †	8(89%) †	14(93%) †
	Disagree	2(8%)	1(11%)	1(7%)
Target of LDL-c <50mg/dL	Agree	16/22(80%) † 1	8/8(100%) † 1	8/14(57%) 1
	Disagree	1/22(5%) 1	0/8(0%) 1	1/14(7%) 1
Target of LDL-c <70mg/dL	Agree	15/22(75%) † 1	4/8(50%) 1	11/14(79%) † 1
	Disagree	4/22(20%) 1	3/8(43%) 1	1/14(7%) 1
Prescribes statin to all patients under secondary prevention	Agree	18(75%) †	6(67%)	12(80%) †
	Disagree	6(25%)	3(33%)	3(20%)
Weighted average of patients that achieved the LDL-c target ( SD )	Statin	50.5%(±20.75)	46%(±19.97%) 3	52.2%(±20.79%) 4
	Statin + Ezetimibe	63.8%(±10.83)*	60.9%(±9.96%)* 3	71.2%(±13.66%)* 4
Would prescribe additional therapy 1 , 2	Yes	18/22(82%)	8/8(100%)	10/14(71%)
	No	4/22(18%)	0/8(0%)	4/14(28%)

† Consensus reached (defined as agreement equal to or greater than 70%);

‡ LDL-c target refers to the treatment goal for patients under secondary prevention; Agree: Strongly agree or agree; Disagree: Disagree or strongly disagree;

1 Anwered only by specialists aiming for an LDL-c target;

2 Administered subcutaneously, indicated for patients who do not reach the target with statin and ezetimibe;

Among patients who do not reach the LDL-c target with statin;

3,4 The weighted average of patients who reach the LDL-c target was calculated based on the consensual targets adopted by each subgroup, considering an LDL-c target of <50 mg/dL for PHS (100% agreement) and <70 mg/dL for SH (79% agreement) for this estimate;

N: Total number of specialists in the Delphi panel;

PHS: Subgroup of specialists from the Public Health System;

SH: Subgroup of specialists from the Supplementary Health System;

**LDL-c:** Low-density lipoprotein cholesterol;

SD: Standard Deviation,

Source: the authors.

### Estimation of the population under secondary prevention

In the PHS, 441,890 individuals with at least one cardiovascular event were identified in 2019. After adjusting for mortality (12.2%) and population growth,^22^the estimated incidence and prevalence for 2024 were 402,615 and 5,822,793 patients under secondary prevention, respectively.

Based on the Delphi panel, it was calculated that 46% (±19.97%) of patients would reach the LDL-c target with statin therapy, while 60.9% (±9.96%) of those who do not reach this target with monotherapy would do so with statin plus ezetimibe. Thus, of the 5,175,816 patients treated by experts who aim for an LDL-c target, it is estimated that 2,380,875 would reach the target with statins, and 1,708,119 would achieve it with statin plus ezetimibe. Therefore, 79% of patients treated by physicians targeting LDL-c would reach the goal. Since all these specialists would prescribe additional therapy for patients not achieving the goal with these lipid-lowering agents, approximately 1,092,822 (19%) patients under secondary prevention in the PHS would be potential candidates for additional LDL-c–lowering therapy ( Figure 2 ).

**Figure 2:** Estimated population eligible for additional pharmacotherapy; estimated population under secondary prevention of cardiovascular diseases based on epidemiological data and the second round of the Delphi panel for the population assisted by the Public Health System – PHS (above) and the Supplementary Health – SH (below), Specialist aiming for LDL-c target: seeks a specific LDL-c goal; Patient not reaching target with statin: patient indicated for combination of ezetimibe and statin; Patient not reaching target with statin and ezetimibe: patient indicated for additional therapy aimed at lowering LDL-c; LDL-c: Low-density lipoprotein cholesterol,

Source: the authors,

In the SH, 81,796 procedures were identified based on the D-TISS panel. After adjusting for mortality and population growth,^23^the estimated incidence and prevalence of individuals with atherosclerotic disease under secondary prevention in 2024 were 82,929 and 1,199,357, respectively.

According to the Delphi panel, 52.2% (±20.79%) of patients would reach the LDL-c target with statins, and 71.2% (±13.66%) of those who do not achieve this target with monotherapy would do so with statin and ezetimibe. Therefore, among the 1,119,400 patients treated by specialists who aim for the LDL-c target, it is estimated that 584,327 would achieve the target with statin therapy, and 380,972 would do so with statin and ezetimibe. In total, 86% of patients treated by physicians who pursue the LDL-c target would reach it. However, 71.4% of specialists would prescribe additional therapy for the 154,101 patients who did not reach the target with statin and ezetimibe, resulting in 110,071 potential candidates for additional LDL-c–lowering therapy in 2024 in the SH ( Figure 2 ).

The probabilistic sensitivity analysis, considering the parameters described in Table 4 , showed that the median and interquartile range of the estimated population eligible for additional lipid-lowering therapy is 1,046,182 (838,337–1,218,850) in the PHS and 118,301 (88,507–150,600) in the SH ( Figure 3 ).

**Table 4:** Parameters for estimating the population under secondary prevention 1 eligible for additional pharmacotherapy

Group	Characteristics	Punctual estimation	Lower limit	Upper limit	Distribution	Source
PHS	Population in secondary prevention 2024	5.822,793	4.658,234	6.987,352	Gama	SIH 2019
	Percentage of experts aiming at the LDL-c target	88.9%	71.1%	100.0%	Beta	Delphi
	Percentage of patients that reach the target with statin	46.0%	26.0%	66.0%	Beta	Delphi
	Percentage of patients that reach the target with ezetimibe	60.9%	50.9%	70.9%	Beta	Delphi
	Percentage of experts that prescribe additional pharmacotherapy *	100%	91.7%	100%	Random	Delphi
SH	Population in secondary prevention 2024	1.199,357	959,86	1.439,228	Gama	D-TISS 2019
	Percentage of experts aiming at the LDL-c target	93.3%	74.7%	100.0%	Beta	Delphi
	Percentage of patients that reach the target with statin	52.2%	31.4%	73.0%	Beta	Delphi
	Percentage of patients that reach the target with ezetimibe	71.2%	57.54%	84.86%	Beta	Delphi
	Percentage of experts that prescribe additional pharmacotherapy *	71.4%	66.7%	93.3%	Beta	Delphi

* aimed at LDL-c reduction;

1 of new cardiovascular events, that do not reach the LDL-c target with statins and ezetimibe;

**LDL-c:** low-density lipoprotein;

PHS: public health system;

SH: supplementary health.

**Figure 3:** Candidates for additional pharmacotherapy to reduce LDL-c; estimated population under secondary prevention of cardiovascular events treated by specialists aiming for the LDL-c target and not reaching the goal with statin and ezetimibe therapy, considered potential candidates for additional LDL-c–lowering pharmacotherapy, based on epidemiological data and the Delphi panel. LDL-c: Low-density lipoprotein cholesterol.

Source: the authors.

## Discussion

The results of the Delphi panel conducted in this study identified that two specialists (7%) did not aim to achieve an LDL-c target – one from each subgroup. Among the others, the LDL-c target of <50 mg/dL, recommended by the SBC,^3^was the goal for specialists in the PHS and for 57% of those in the SH; in the latter, consensus was reached for a target below 70 mg/dL.

Contrary to SBC recommendations, the Ministry of Health’s Clinical Protocol and Therapeutic Guidelines for Dyslipidemia recommend aggressive lipid-lowering therapy without a specific target for patients under secondary prevention.^4^These discrepancies are also observed in the medical literature.^28^Between 2015 and 2020, studies involving very high-risk patients more frequently used an LDL-c reference target of <70 mg/dL,^29 - 38^while those published after 2021 adopted a lower target of 55 mg/dL.^39 - 41^

The Delphi panel indicated that statins are prescribed to more than 80% of patients under secondary prevention, similar to the REACT study,^42^which showed statin prescription for 77.7% of outpatients under secondary prevention in the PHS or SH.^42^However, this is higher than the percentage of high cardiovascular risk patients who reported using lipid-lowering agents (55.2%) at the beginning of the ELSA-Brasil study.^43^

Regarding the statin prescribed, a retrospective study conducted at a Brazilian hospital showed that simvastatin (77.6%) and atorvastatin (22.4%) were most frequently prescribed for the population assisted by the PHS.^44^In this study, simvastatin was also most reported in the PHS, while rosuvastatin prevailed in the SH. The reason for this difference could not be clearly established, but the free provision of simvastatin and atorvastatin by SUS^45^and the high cost of rosuvastatin may partly explain these findings.

The difference in the percentage of patients who achieve the target in the PHS and SH may be related to discrepancies in the targets reported by specialists or to the potency of the statins used. In addition, socioeconomic differences and access to healthcare may contribute to better LDL-c control in the SH, as evidenced in the ELSA-Brasil study, where LDL-c control was more frequent in the SH (62.4%) compared to the PHS (45.6%).^43^However, when specifically analyzing patients under secondary prevention, a retrospective study estimated that only 7.4% of patients assisted in the PHS had LDL-c levels <50 mg/dL and 28.9% <70 mg/dL.^12^

Among the specialists aiming for an LDL-c target, all in the PHS and 71.4% in the SH would prescribe additional pharmacotherapy for patients who did not reach the recommended goal with lipid-lowering treatment—a result similar to another study in which 80% of specialists would prescribe additional therapy.^46^The more common prescription in the PHS may reflect the clinical complexity of the patients assisted, in addition to the free provision of medications. In contrast, in the SH, the high cost of medications used by patients under secondary prevention may be considered a limiting factor.

The percentage of patients eligible for additional therapy in the SH is consistent with estimates by Cannon et al.,^47^who estimated that 86% of patients under secondary prevention treated with statins, alone or in combination with ezetimibe, would reach LDL-c levels <70 mg/dL, while 14% would require additional therapy.^47^Similarly, Virani et al.^48^estimated that 24.5% of patients under secondary prevention treated with statins and ezetimibe would be candidates for additional therapy – a percentage similar to that found in the PHS.^48^These data reinforce that, although statins and ezetimibe are effective in reducing LDL-c, many patients may benefit from additional therapies to achieve the LDL-c target recommended by SBC guidelines.^3^Currently, three technologies are registered for this indication in Brazil: alirocumab, evolocumab,^3^and inclisiran.^49^

Among the limitations of the study, the inability to estimate the percentage of patients who achieve the target by type of statin stands out, as the potency of the drugs varies. The absence of patient interviews prevented the assessment of adherence to lipid-lowering therapy and willingness to use additional subcutaneous therapies. Furthermore, assuming that each procedure corresponds to a cardiovascular event (incidence) may underestimate the population under secondary prevention, since patients without acute events but diagnosed in outpatient settings (e.g., stable angina) were not included. However, given the complex nature of these events, the impact is likely minimal. External validity is limited due to the predominance of specialists from Rio de Janeiro, which restricts the national generalization of the results because of regional variations in medical practices and targets adopted. Other limitations inherent to the Delphi panel include the use of a convenience sample, sample size, and the reduction in the number of participants between rounds. Despite these limitations, prospective studies assessing success rates in achieving LDL-c targets in real-life populations, as well as cost-effectiveness analyses of additional therapies in the SUS and SS, may contribute to improving dyslipidemia management in Brazil.

## Conclusion

Based on the analysis conducted in this study, it is estimated that in 2024, approximately six million Brazilians will be under secondary prevention of cardiovascular events in the PHS and around one million in the SH (Central Figure). Moreover, it is believed that between 9% and 19% of these patients will not reach the LDL-c target recommended by current medical guidelines and could benefit from additional LDL-c–lowering therapy to reduce the risk of new cardiovascular events.

Data Availability

The underlying content of the research text is contained within the manuscript.

## *Supplemental Materials

For additional information, please click here.
